# Exploring biochar and fishpond sediments potential to change soil phosphorus fractions and availability

**DOI:** 10.3389/fpls.2023.1224583

**Published:** 2023-08-10

**Authors:** Mohsin Mahmood, Yunting Wang, Waqas Ahmed, Sajid Mehmood, Anam Ayyoub, Ahmed S. M. Elnahal, Weidong Li, Xin Zhan

**Affiliations:** ^1^ Key Laboratory of Agro-Forestry Environmental Processes and Ecological Regulation of Hainan Province, Hainan University, Haikou, China; ^2^ Center for Eco-Environment Restoration Engineering of Hainan Province, Hainan University, Haikou, China; ^3^ College of Life Sciences, Northwest A&F University, Yangling, Shaanxi, China; ^4^ Pathology Department, Faculty of Agriculture, Zagazig University, Zagazig, Egypt; ^5^ State Key Laboratory of Marine Resource Utilization in South China Sea, College of Marine Science, Hainan University, Haikou, China

**Keywords:** fishpond sediments, metal contents, biochar, P uptake, phosphorus fractions, Tiessen and Moir fractionation scheme

## Abstract

Phosphorus (P) availability in soil is paradoxical, with a significant portion of applied P accumulating in the soil, potentially affecting plant production. The impact of biochar (BR) and fishpond sediments (FPS) as fertilizers on P fixation remains unclear. This study aimed to determine the optimal ratio of BR, modified biochar (MBR), and FPS as fertilizer replacements. A pot experiment with maize evaluated the transformation of P into inorganic (Pi) and organic (Po) fractions and their contribution to P uptake. Different percentages of FPS, BR, and MBR were applied as treatments (T1–T7), T1 [(0.0)], T2 [FPS (25.0%)], T3 [FPS (25.0%) + BR (1%)], T [FPS (25%) +MBR (3%)], T5 [FPS (35%)], T6 [FPS (35%) +BR (1%)], and T7 [FPS (35%) + MBR (1%)]. Using the modified Hedley method and the Tiessen and Moir fractionation scheme, P fractions were determined. Results showed that various rates of MBR, BR, and FPS significantly increased labile and moderately labile P fractions (NaHCO_3_-P_i_, NaHCO_3_-P_o_, HCl_D_-P_i_, and HCl_C_-P_i_) and residual P fractions compared with the control (T1). Positive correlations were observed between P uptake, phosphatase enzyme activity, and NaHCO_3_-Pi. Maximum P uptake and phosphatase activity were observed in T6 and T7 treatments. The addition of BR, MBR, and FPS increased Po fractions. Unlike the decline in NaOH-Po fraction, NaHCO_3_-Po and HClc-Po fractions increased. All Pi fractions, particularly apatite (HCl_D_-Pi), increased across the T1–T7 treatments. HCl_D_-P_i_ was the largest contributor to total P (40.7%) and can convert into accessible P over time. The T5 treatment showed a 0.88% rise in residual P. HCl_D_-P_i_ and residual P fractions positively correlated with P uptake, phosphatase activity, NaOH-Pi, and NaOH-Po moderately available fractions. Regression analysis revealed that higher concentrations of metals such as Ca, Zn, and Cr significantly decreased labile organic and inorganic P fractions (NaHCO_3_-Pi, *R* ^2 = ^0.13, 0.36, 0.09) and their availability (NaHCO_3_-Po, *R* ^2 = ^0.01, 0.03, 0.25). Excessive solo BR amendments did not consistently increase P availability, but optimal simple and MBR increased residual P contents in moderately labile and labile forms (including NaOH-Pi, NaHCO_3_-Pi, and HCl_D_-Pi). Overall, our findings suggest that the co-addition of BR and FPS can enhance soil P availability via increasing the activity of phosphatase enzyme, thereby enhancing plant P uptake and use efficiency, which eventually maintains the provision of ecosystem functions and services.

## 1 Introduction

Phosphorus (P) is a viable mineral resource for crop growth development in agriculture (Rubæk et al., 2013; Wei et al., 2017). About 50–70% of the total P in soils is non-labile and cannot be taken by plants (Mahmood et al., 2020). According to Wang et al. (2023), soil presence of different metal ions and nutrients cause the loss of applied P by fixation. Therefore, incorporating efficient adsorbents in soil can maintain the level of P availability by plants, and simultaneously reduce its fixation.

There are various forms of P in soil, including organic (Po) and inorganic (Pi), which may be divided into labile, non-labile, and moderately labile P fractions (Redel et al., 2008; Gatiboni and Condron, 2021). By various soil processes (biological, physiological, and chemical), applying various absorbents in soil may change both P fractions’ distribution in the soil (Damian et al., 2020). For instance, using biochar (BR) might influence microbial activities, which in turn leads to the mineralization of the Po and Pi fractions (Li et al., 2020). While the utilization of soil fishpond sediments (FPS) as fertilizer can potentially increase the content of Po by influencing primary production and stimulating the demand for (Pi) in plants (Choi et al., 2015).

In recent years, soil P has been extensively explored using diverse adsorbents, including fly ash (Zhao et al., 2019), crop residue (Noack et al., 2012), and other materials. However, these adsorbents have drawbacks, such as a weak adsorption capacity and a decrease in P availability (Mehmood et al., 2020; Yu et al., 2021). A type of persistent, refractory, strongly aromatic, and carbon-rich solid material known as “biochar” is created by the gradual, relatively low-temperature pyrolysis of biological leftovers in a low-oxygen environment (Wang and Wang, 2019; Qiu et al., 2022). Based on the studies that have been reported, various mechanisms have been identified to account for the adsorption of Po by BR. One mechanism involves complexation, where metal ions inside the solution are more easily bound to the phosphate groups when metal oxides or hydroxides, such as Ca (OH)_2_, MgO, and Fe_2_O_3_, are present on the surface of the BR (Zhang et al., 2018; Wu et al., 2020; Peng et al., 2021).

FPS are abundant in nutrients and trace elements and can potentially serve as beneficial fertilizers for promoting crop growth (Al-Solaimani et al., 2022). Although it has been widely researched how nutrients may build up in sediments (Schmadel et al., 2019; Becker and Silbiger, 2020; Huang et al., 2022), the collected sediments are unsuitable for direct application due to the preservation of metal contents on their particle surfaces (Gao et al., 2021). To ensure the safe usage of FPS as fertilizers in agricultural soils, sediments must be converted into environment friendly source of fertilizer (Yan et al., 2018).

Despite the abundance of P in the soil, a significant portion becomes bound to mineral surfaces or transformed into recalcitrant forms, leading to limited bioavailability for plants (Ikhajiagbe et al., 2020). This phenomenon is particularly common due to different metals contents like Ca, Fe, Cd, Mg, and Zn, which can precipitate and interact with P (Ogut et al., 2010; Yami et al., 2016; Sylvia et al., 2021). Nonetheless, P is a crucial constituent of numerous essential biochemical compounds, including nucleic acids, phospholipids, amino acids, and ATP, and it plays a critical role in promoting plant growth (Campos et al., 2018; Srivastava et al., 2018). Providing a well-balanced P supply not only supports the development and growth of crops but also motivates plants to enhance their strategies for effective P uptake and utilization, ensuring their survival (Yan et al., 2023).

Phosphatase plays a critical role in facilitating the conversion of (Po) into bioavailable (Pi), specifically phosphate, in soil (Mahmood et al., 2022). It catalyzes the hydrolysis of esters and anhydrides of phosphoric acid, thereby enhancing the P bioavailability (Qin et al., 2022). It is important to recognize that phosphatase activity plays a crucial role in plant nutrient acquisition and is also exceptionally responsive to metal concentrations. In fact, its activity has been utilized as an effective measure for assessing soil P availability (Foster et al., 2018; Pokharel et al., 2020). Hence, it is imperative to examine the P acquisition strategies employed by plants in response to the lower-P conditions resulting from the usage of BR for remediating excessive metal content in soil. Moreover, it is crucial to identify BR types that can prevent the occurrence of P deficiency in the soil.

Recent investigations have demonstrated that modified biochar (MBR) can serve as an innovative and efficient solution to improve soil P use efficiency and plant uptake by lowering the bioavailability of specific elements, such as cadmium (Cd) and calcium (Ca), in the soil (Ahmed et al., 2023). According to Gonzaga et al. (2022) and Ahmed et al. (2023), utilizing MBR holds the potential to serve as a valuable resource for both energy and nutrient provision, increasing the amount of accessible P in the soil and giving plants nutrients. Although considerable study has been done on P-MBR, it is still unknown if this BR can be incorporated with sediments can prevent relative P shortages in soils.

Therefore, this study is to investigate the impact of BR and fishpond sediments on P distribution and availability status in soil. We hypothesized that (1) the addition of BR and FPS would increase P availability and reduce its fixation, which can hinder soil P supply; (2) solo BR application would stimulate soil P uptake by plants, potentially triggering an increase in phosphatase release to ensure an adequate P supply; (3) MBR with FPS could promote uptake by increasing soil P availability from legacy present in soils.

## 2 Materials and methods

### 2.1 Preparation of biochar-amended fishpond sediments

The soil under investigation was supplemented with sediment samples collected from a fishpond located in Tan Niu, China. The FPS were mixed with 3.0% (w/w) BR obtained from Taraxacum mongolicin, a Chinese herb. The BR was produced in a tube furnace heated at 7°C per min until it reached 500°C and was maintained at that temperature for 1h. Subsequently, the mixture was incubated under constant temperature conditions in the absence of light for a duration of 90 days, as described in the study by Mehmood et al. (2023). In this study, FPS will be referred as FPS, while BR-treated FPS will be referred as MBR.

### 2.2 Soil sampling and experimental setup

This study was carried out during 2021–2022 under greenhouse conditions in the Department of Ecology and Environment, Hainan University, Hainan, China. Topsoil (0–15 cm) was collected from farmland near Haikou, China (20° 03′ 22.80′′ N and 110° 19′ 10.20′′ E). The soil was air-dried in a ventilated dry and in-shadow place for a week. Then, plant debris were removed, and the soil was ground, 2-mm sieved and properly stored for subsequent analysis and experiments. The basic properties of soil, FPS, BC, and MBR are presented in **Table 1**.

**Table 1:** Basic attributes of the soil and fishpond sediments (FPS), *Taraxacum mongolicum* Hand-Mazz derived biochar (BR), and biochar-treated sediments (MBR).

Parameters	Unit	Soil	FPS	BR	MBR
pH		5.07	6.06	8.91	6.19
EC	mS m^–1^	6.70	14.50	60.00	15.35
Total N	g kg^–1^	0.63	1.59	1.98	1.73
Available P	mg kg^–1^	1.00	103.00	147.40	163.55
Exchangeable K	g kg^–1^	0.60	1.28	1.97	1.32
Total Ca	g kg^–1^	1.00	1.60	2.51	1.81
Total Mg	g kg^–1^	1.40	2.10	0.41	2.23
Total Cr	mg kg^–1^	57.00	38	n.d.	27.98
Total Zn	mg kg^–1^	64	28	n.d	n.d
Total Cd	mg kg^–1^	0.043	0.064	n.d	n.d

not detected denoted by n.d.

The treatments were added to the soil on w/w basis with the dose of T1 [(0.0)], T2 [FPS (25.0%)], T3 [FPS (25.0%) + BR (1%)], T4 [FPS (25%) + MBR (3%)], T5 [FPS (35%)], T6 [FPS (35%) + BR (1%)], and T7 [FPS (35%) + MBR (1%)]; each soil treatment was placed into plastic pots (containing 5 kg of air-dry soil). Maize (sweet glutinous 3000) seeds were acquired from Chun Xia Qiu Dong Zhong Ye (Hengyang, Hunan, China). Healthy seeds were disinfected for 5 min with 5% (w/v) NaOCl solution, and then 10 seeds were sown in each plastic pot containing a mixture of soil, FPS, and MBR. After germination, maize seedlings were thinned to five healthy seedlings per pot. Plants were irrigated according to the plants’ needs (typically three times per week). Seedlings without FPS and MBR were used as the control plants. The experimental pots were organized in a randomized complete block (RCB) design, with three replicates. The average day/night temperatures during the experiment were 21/27°C ( ± 3°C), and relative humidity was 65 ± 5%.

### 2.3 Soil and plant analysis

Maize plant samples were collected after 40 days of sowing. The plants were carefully harvested by gently detaching them from each pot by hand, and the roots were promptly separated from the stem–root junction. The aboveground portions from each pot were combined to measure P uptake. Subsequently, the plants were air-dried initially and then subjected to 30 min of oven drying at 90°C to halt all metabolic activities. This was followed by further drying at 65°C for 48h to determine the extent of total plant P content. P uptake was calculated from the measured concentration and plant biomass.

To measure the phosphatase activity, the method proposed by Eivazi and Tabatabai (1977) was used. One gram of soil was treated with 0.25 mL of toluene. Subsequently, a solution of 4 mL of a universally modified buffer and 1 mL of a p-nitrophenyl phosphate solution were introduced to this buffer and incubated for 1h at 37°C. The universally modified buffer was prepared by combining 14 g of citric acid, 12 g of Tris, 11.6 g of maleic acid, 6.3 g of boric acid, 500 ml of NaOH, and 1000 ml of dH_2_O were combined. After the incubation period, 4 mL of (NaOH, 0.5 M) and 1 mL of (CaCl_2_, 0.5 M) have been added. The absorbance at 400 nm was determined after combining and filtering all the components with (Whatman no. 42), and the absorbance was measured using spectrophotometer. The potential phosphatase activity is expressed as µg pNP g^−1^ h^−1^.

The concentrations of (Ca), (Mg), (Cd), (Zn), and chromium (Cr) were determined using standard analytical techniques “NY/T 296-1995.” For Ca and Mg analysis, samples were typically extracted using acid digestion methods, using of dilute hydrochloric acid (HCl). The extracted solutions were then analyzed using atomic absorption spectrometry (AAS). Similarly, Zn, Cd, and Cr concentrations were measured using ICP-AES after appropriate extraction methods, such as acid digestion or sequential extraction.

#### 2.3.2 Measurement of soil P fractions

A modified P fractionation scheme, initially established by Hedley et al. (1982) and later modulated by Tiessen and Moir, was employed to sequentially extract different soil P fractions (Mahmood et al., 2021). For the extraction of NaHCO_3_-Pi, HCl_D_-Pi extractable P fractions, NaOH-Pi, and 1 g of soil was subjected to extraction with 0.1 M NaOH, 30 ml of 0.5 M NaHCO_3_ (pH 8.5), and 1 M dilution HCl (HCl_D_-P_i_), respectively, for a duration of 16h each. Additionally, the remaining soil was further extracted with 15 mL of 1 M concentrated HCl (HCl_C_-Pi) at (80°C) for 20 min using a hot water bath. Residual P was determined using 5 mL H_2_SO_4_ and H_2_O_2_ at 360°C. Inorganic P (Pi) were determined from the filtrates of NaOH^−^, HClc-extractable P, and NaHCO_3_ by dividing them into two groups of samples. For NaOH-Pi and NaHCO_3_-Pi, 0.9 M H_2_SO_4_ was introduced to the filtrate, followed by centrifugation at 4000 rpm at 0°C for 10 min prior to analysis. HCl_C_-Pi, on the other hand, was directly assessed without any pretreatment. For The Pi, the determination of HCl_D_-Pi filtrates and residual P was carried out directly. UV spectrophotometer was used for all P analyses (Pi). The (Po) fraction was determined by calculating the discrepancy between the concentrations of the total P (Pt) and (Pi). The total P was computed as the aggregate of organic and inorganic P fractions (Mirabello et al., 2013).

### 2.5 Statistical analysis

Data analysis was conducted using the one-way (ANOVA) analysis to determine significant differences among treatments. Treatment means were compared using the Least Significant Differences (LSD) test at a significance level of *p* = 0.05, utilizing SPSS 21. by the test of least significant differences (LSD) at *p* = 0.05 using SPSS 21. Pearson correlation analysis was performed to assess the relationship between different P fractions, metal contents, P uptake and phosphatase enzyme activity using R software. We used regression analysis to determine changes in P fractions, by the distribution of different metal contents.

## 3 Results

### 3.1 P uptake and phosphatase enzyme activity

**Figure 1:**
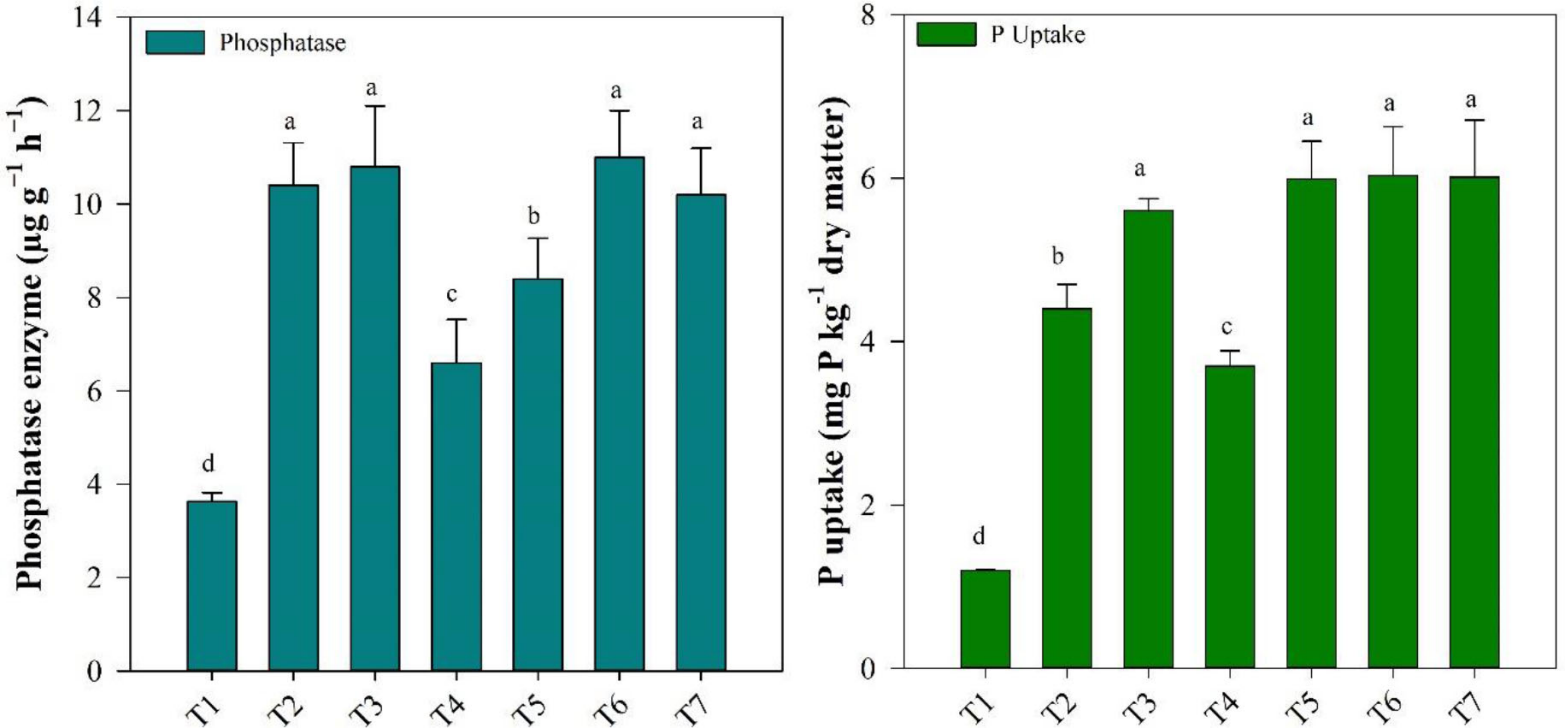
Response of phosphatase enzyme activity and P uptake to fishpond sediments (FPS), biochar (BR), and modified biochar (MBR) treatments. T1 [(control)], T2 [FPS (25.0%)], T3 [FPS (25.0%) + BR (1%)], T [FPS (25%) +MBR (3%)], T5 [FPS (35%)], T6 [FPS (35%) + BR (1%)], and T7 [FPS (35%) + MBR (1%)] applications. Bars over the marker show standard error (*n* = 3). Different letters above the columns indicate significant differences at *p* < 0.05.

The application of FPS, BR, and/or MBR changed the soil phosphatase activities to varying degrees (**Figure 1**). The phosphatase enzyme activity was significantly lower in T4 and T5 treatments (6.6 and 8.4 µg pNP g^−1^ h^−1^), respectively, than in other applied treatments of BR and FPS (*P* < 0.05). A higher but non-significant difference was found in phosphatase activity among the treatments T2, T3, T6, and T7, respectively.

The mean value of P uptake ranged from 1.3 to 6.03 mg P kg^−1^ dry matter. A higher P uptake (6.03 mg P kg^−1^ dry matter) was found in the T6 treatment, which increased by 5.79% compared with T1 treatment. However, P uptake showed no significant difference among T3, T5, T6, and T7, while it was significantly lower T4 and T2 as compared with the T1 treatment (3.7, 4.4, and 1.2 mg P kg^−1^ dry matter) (**Figure 1**).

### 3.2 Residual P fraction and Total P

**Figure 2:**
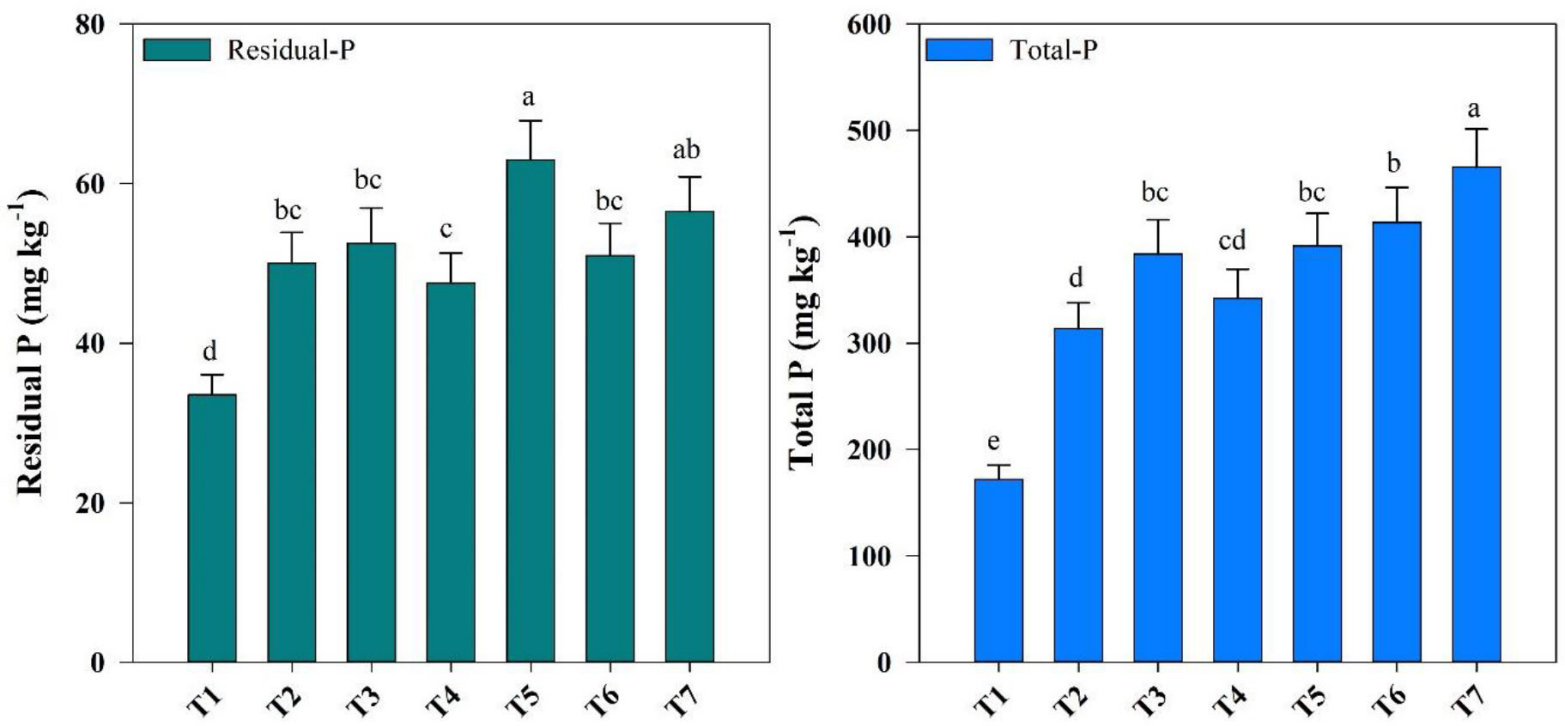
Changes in residual P and total P in response to different fishpond sediments (FPS), biochar (BR), and modified biochar (MBR) treatments. Treatments include T1 [(0.0)], T2 [FPS (25.0%)], T3 [FPS (25.0%) + BR (1%)], T [FPS (25%) + MBR (3%)], T5 [FPS (35%)], T6 [FPS (35%) + BR (1%)], and T7 [FPS (35%) + MBR (1%)]. Error bars show the standard error. Different letters above the columns indicate significant differences at *p* < 0.05.

Residual P concentration was significantly affected by the combined and solo application of BR and FPS (**Figure 2**); compared with the control treatment (T1) (33.5 mg kg^-1^), residual P was 0.88% and 0.68% higher in T5 and T7 treatment (63.00 mg kg^-1^ and 56.50), respectively, while stagnated among the other treatments.

Similarly, the response of total P showed an increasing trend and ranged from 171.90 to 465.34 mg kg^−1^. The highest total P was recorded in T7 treatment (465.34 mg kg^−1^). Compared with the control treatment (T1), it was recorded 1.40 and 1.70% higher in T6 and T7 treatments (**Figure 2**).

### 3.3 Organic P fractions

**Figure 3:**
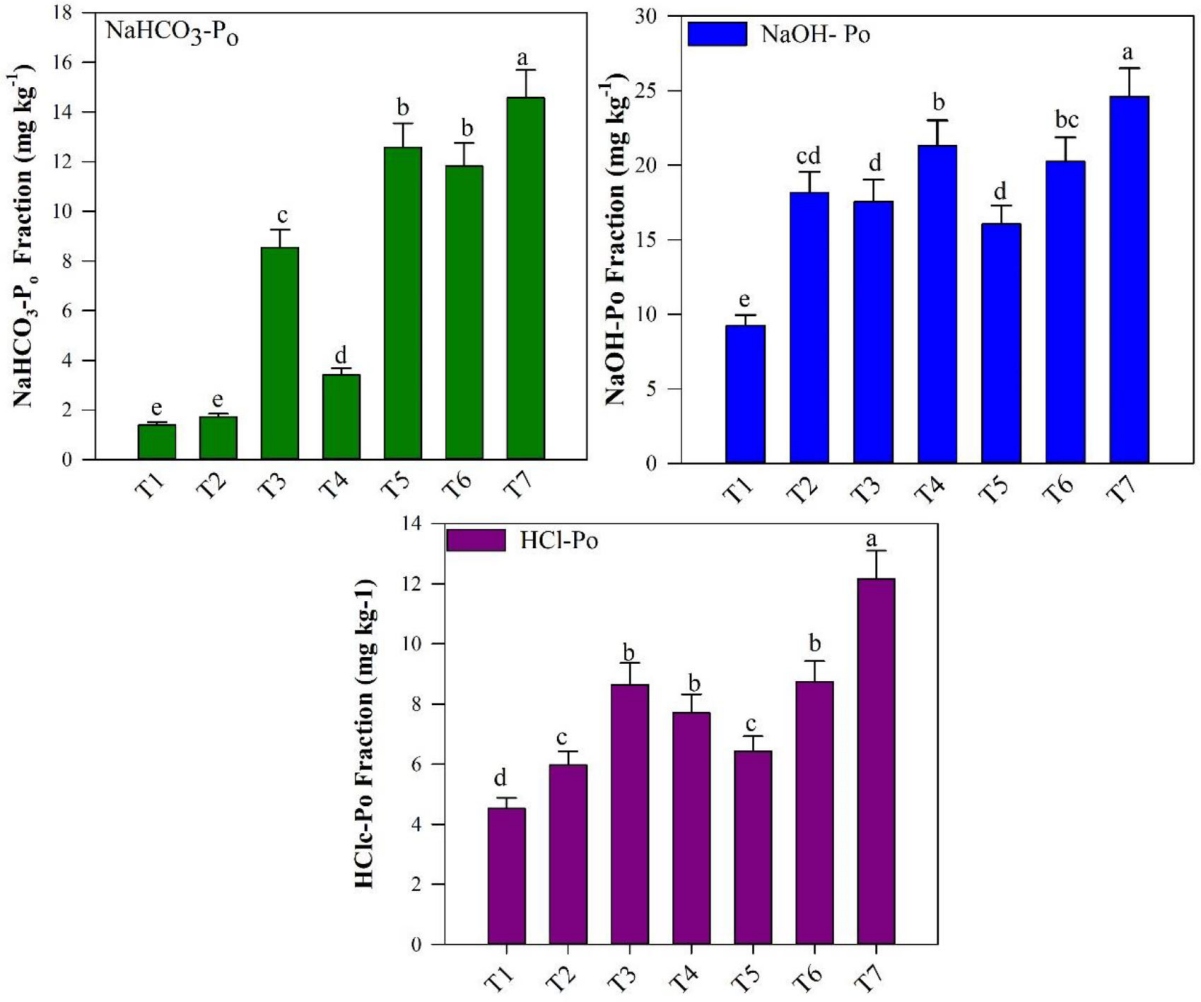
Changes in organic P fractions (NaHCO_3_-P_o_, NaOH-P_o_, and HCl_C_-P_o_) in response to different fishpond sediments (FPS), biochar (BR), and modified biochar (MBR) treatments. Treatments include T1 [(0.0)], T2 [FPS (25.0%)], T3 [FPS (25.0%) + BR (1%)], T [FPS (25%) + MBR (3%)], T5 [FPS (35%)], T6 [FPS (35%) + BR (1%)], and T7 [FPS (35%) + MBR (1%)]. Bars over the marker show standard error (n = 3). Different letters above the columns indicate significant differences at *p* < 0.05.

The organic P fractions (NaHCO_3_-P_o_ and HClc-P_o_) were significantly influenced by the BR and FPS amendments (**Figure 3**), while NaOH-P_o_ showed lower concentration among the treatments. The mean value of NaHCO_3_-P_o_ was 11.82 mg kg^−1^ and 14.57 mg kg^−1^ in T6 and T7 treatments, while HCl-P_o_ value was recorded significantly higher in T7 (12.17 mg kg^−1^) while lower in other treatments. Compared with the control treatments, NaHCO_3_-P_o_ and HCl-P_o_ increased by 4.07 and 1.66% in T7 treatments. However, HCl-P_o_ increased by 1.68% in T7 treatment as compared with the control (T1).

### 3.4 Inorganic P fractions

**Figure 4:**
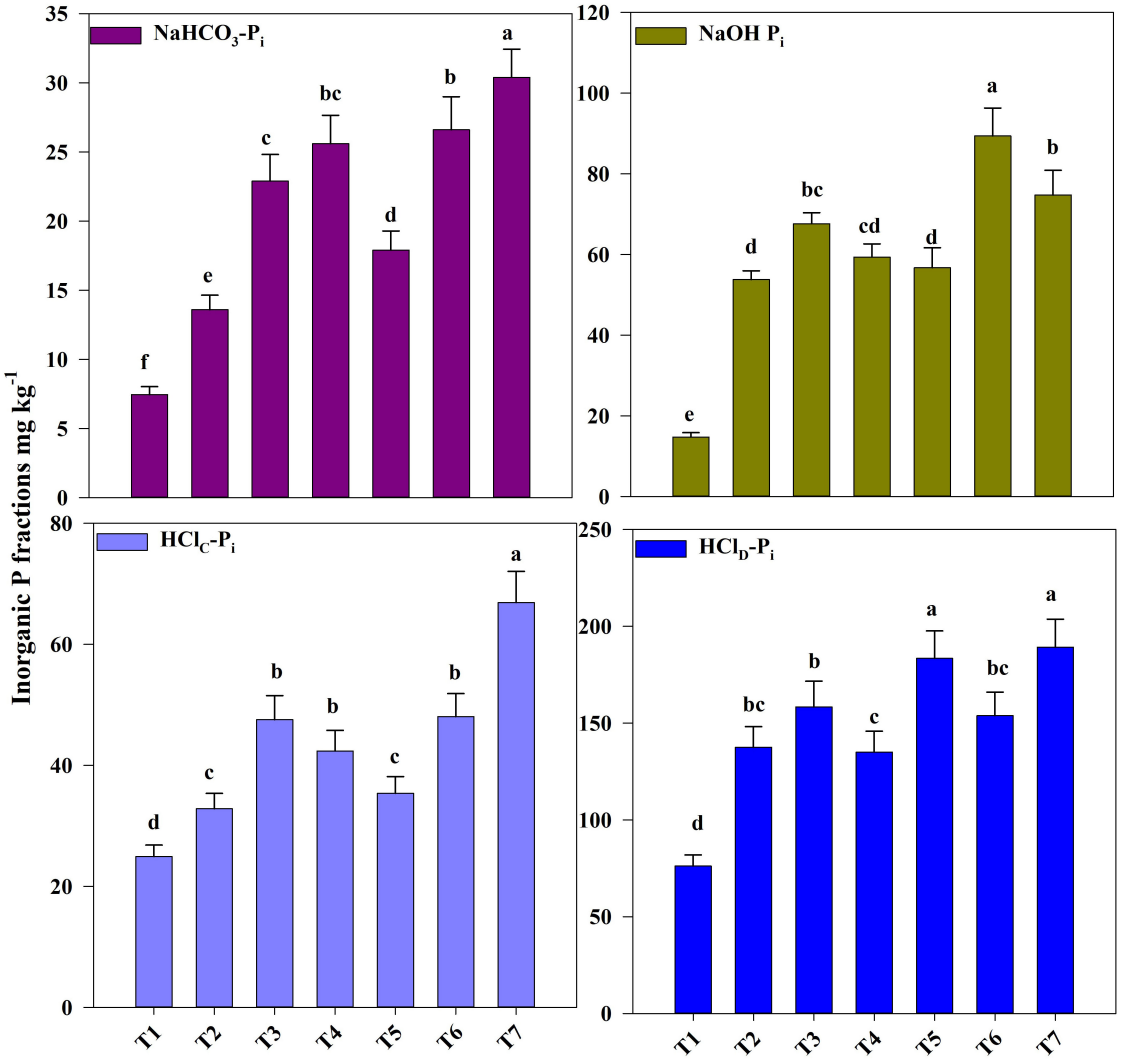
Changes in inorganic *P* fractions (NaHCO_3_-P_i_, NaOH-P_i_, HCl_D_-P_i_, and HCl_C_-P_i_) in response to different fishpond sediments (FPS), biochar (BR), and modified biochar (MBR) treatments. Treatments include T1 [(0.0)], T2 [FPS (25.0%)], T3 [FPS (25.0%) + BR (1%)], T [FPS (25%) + MBR (3%)], T5 [FPS (35%)], T6 [FPS (35%) + BR (1%)], and T7 [FPS (35%) + MBR (1%)]. Bars over the marker show standard error (n = 3). Different letters above the columns indicate significant differences at *p* < 0.05.

FPS and BR variables application significantly increased the proportion of various P_i_ fractions (**Figure 4**) including NaHCO3-Pi, NaOH-P_i_, HCl_D_-P_i_, and HCl-P_i_, respectively, and ranged from 7.46 mg kg^−1^ to 30.40, 14.76 to 89.42 mg kg^−1^, 76.09 to 189.21 mg kg^−1^, and 24.92 to 66.93 among the treatments. However, higher concentration of NaHCO_3_-Pi (30.40) and NaOH-Pi (89.42) were found in T7 and T6 treatments, respectively, while HCl_D_-Pi and HClc-Pi fraction were significantly higher in T7 treatment. Among all the inorganic fractions, HC_D_-Pi was the highest contributor (37.2–46.9%) in the total P in all study treatments (**Figure 4**).

### 3.5 Response of P fractions to Ca, Mg, Cr, Zn, and Na

**Figure 5:**
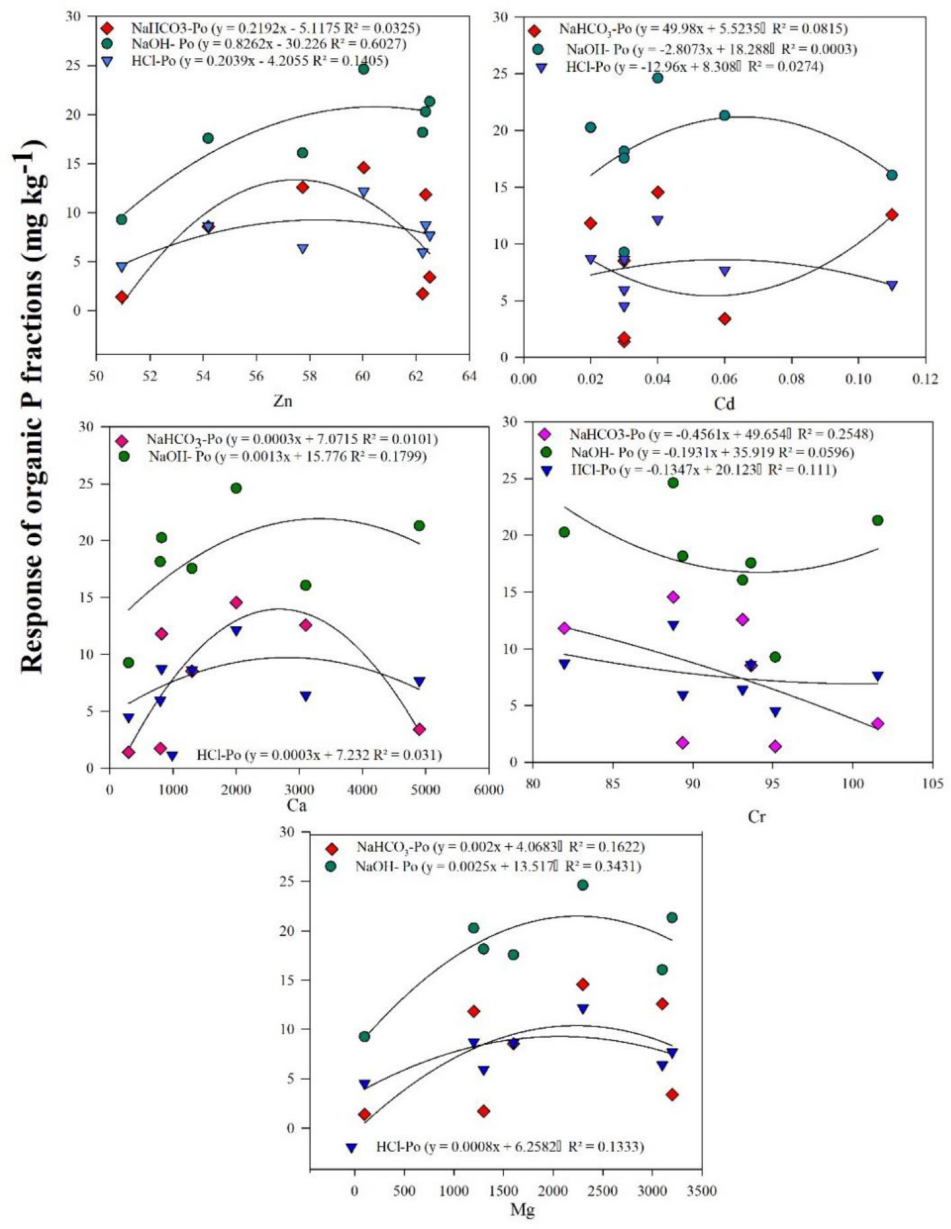
Regression analysis showing the significant changes in organic P fractions (NaHCO_3_-P_o_, NaOH-P_o_, and HCl_C_-P_o_) response to different metal contents distribution (Zn, Cd, Ca, Cr, and Mg).

We have computed regression analysis to measure the response of organic P fractions (Po) to Ca, Mg, Cr, Zn, and Na (**Figure 5**). Our linear regression showed the significant increasing response of NaOH -Po (*R* ^2^ = 0.60*; *p* < 0.05) and HClc-Po (*R* ^2^ = 0.14, *p* > 0.05) to the increase in Zn concentration in HClc-Po and, respectively, while decreasing trend found in NaHCO_3_-Po with the increase in Zn concentration (*R* ^2^ = 0.032, *p* < 0.05; **Figure 5**). Likewise, higher Cd concentrations increased the concentration of labile organic P (NaHCO_3_-Po, *R* ^2^ = 0.081) while NaOH (*R* ^2^ = 0.081) and HCl-Po (*R* ^2^ = 0.0003) fractions stagnated. Ca concentration non significantly affected the distribution of labile organic P and showed decreasing trend with the increase in Ca content (*R* ^2^ = 0.010). However, non-significant change was observed in NaOH and HClc-Po with the increase in Ca concentration (**Figure 5**). Cr concentration significantly decreased NaHCO_3_-Po and (*R* ^2^ = 0.25) and increased the NaOH-Po with increase in its concentration (*R* ^2^ = 0.059); however, HClc-Po showed non-significant change in response to Cr (**Figure 5**). Mg concentration increased the all-organic P fractions (NaHCO_3_-P_o_, NaOH-P_o_, and HCl_c_-P_o_) with increase in its concentration.

**Figure 6:**
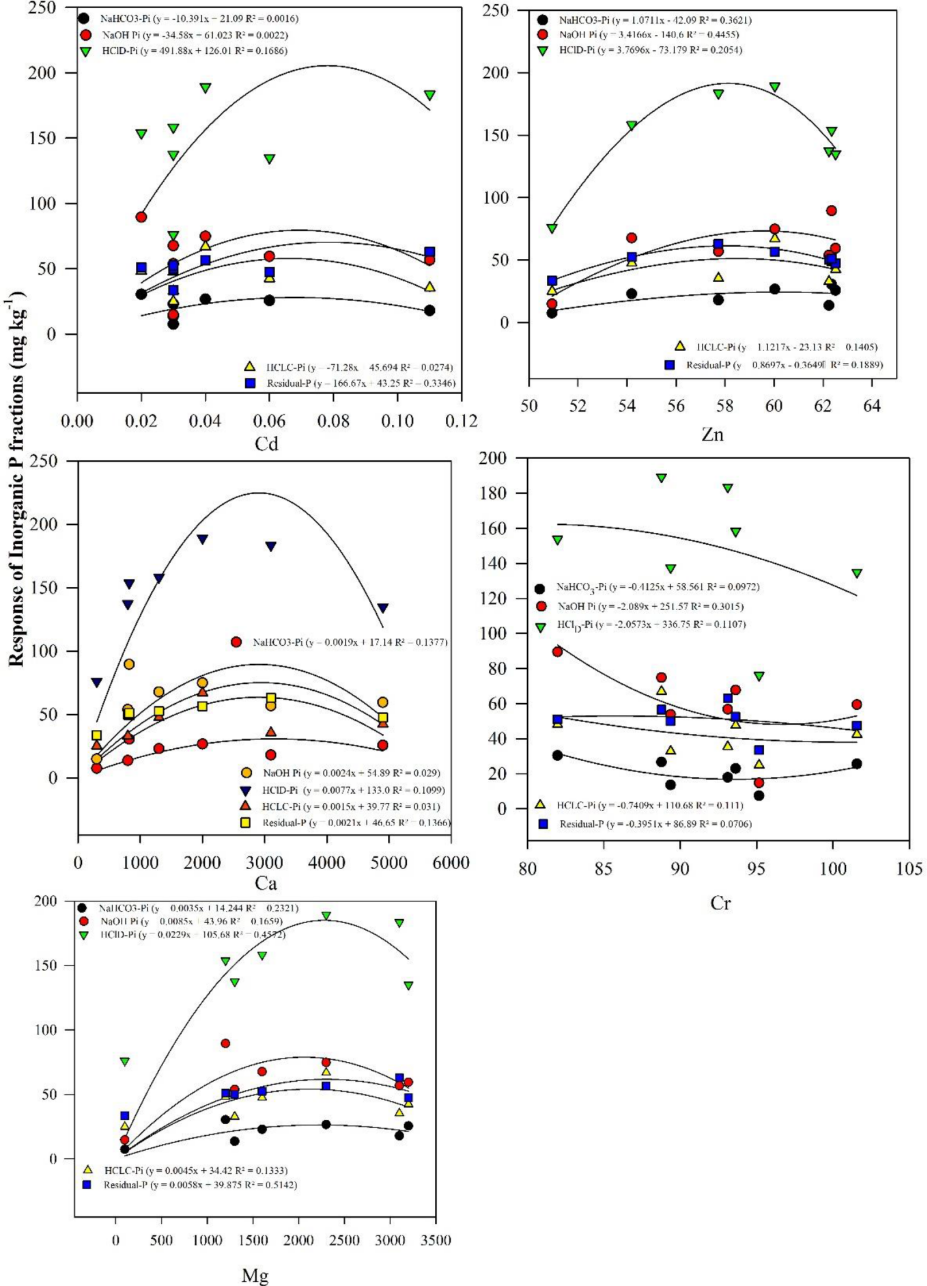
Regression analysis showing the significant changes in inorganic P fractions (NaHCO_3_-P_i_, NaOH-P_i_, HCl_D_-P_i_, HCl_C_-P_i_, and residual P) response to different metal contents distribution (Zn, Cd, Ca, Cr, and Mg).

Similarly, linear regression showed the quantitative variation in different P_i_ fractions response to Cd, Zn, Ca, Cr, and Mg concentrations (**Figure 6**, *p* < 0.05). Proportion of inorganic fractions in response to Cd concentration was found in the order of HCl_D_-P_i_>Residual-Pi> NaOH-P_i_> HCl_c_-P_i>_ NaHCO_3_-P_i_ (*R* ^2^ = 0.16, 0.33, 0.022, 0.024, and 0.40, respectively (**Figure 6**). Likewise, increase Zn concentration showed no change in inorganic P fractions except HCl_D_-Pi fraction (*R* ^2^ = 0.20) which was decreased with increase in Zn concentration. In response to increase Ca concentration HCl_D_-Pi (*R* ^2^ = 0.10) showed maximum decrease as compared with other inorganic P fractions. Similar trends were observed in response to Cr and Mg Concentration in soils, HCl_D_-Pi (*R* ^2^ = 0.11 and 0.45), respectively (**Figure 6**).

### 3.8 Pearson correlation

**Figure 7:**
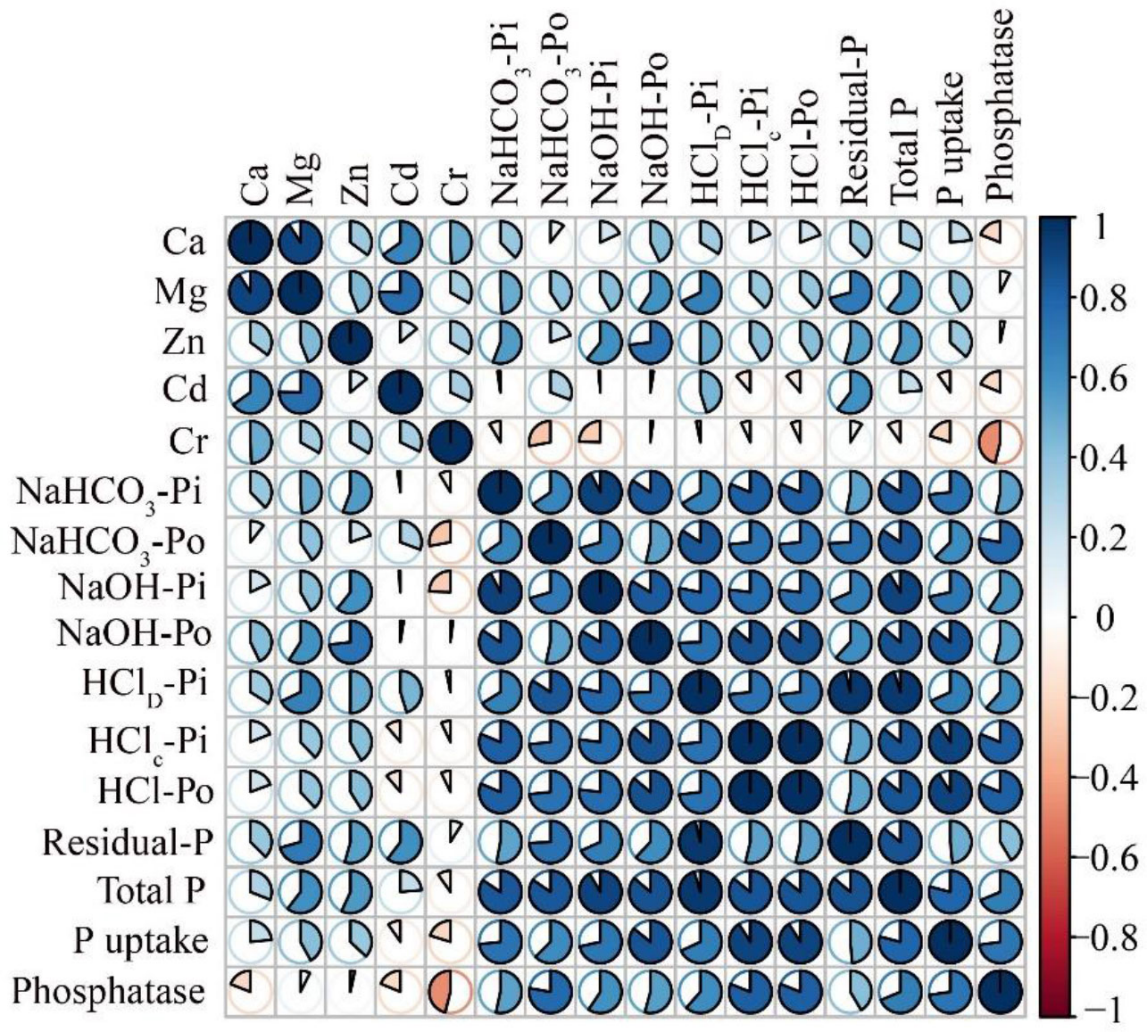
Pearson’s correlation matrix between P fractions (inorganic and organic), metal contents (Zn, Cd, Ca, Cr, and Mg), phosphatase enzyme activity and P uptake under FPS, biochar (BR) and modified biochar (MBR) applications. Correlations are displayed in blue (positive) and red (negative).

The relationship between various P fractions, P uptake, and metal contents was evaluated using a Pearson correlation coefficient (**Figure 7**). We observed a strong positive correlation between NaHCO_3_-Pi, HCl_D_-Pi, and Ca being linked with the P uptake and phosphatase enzyme activity under different variables of BR-, MBR-, and FPS-incorporated soils. NaOH-Pi showed a strong positive correlation with total P and phosphatase activity and HCl-Po and Mg and Zn. Fractions of P responses (NaHCO_3_-P_o_, NaOH-P_i_, P_o_, HClc-Pi, and total P) and different elements (Cd, Cr, and Zn) pose a strong positive correlation between them. Residual-Pi were significantly and positively correlated with phosphatase and total P uptake. (**Figure 7**).

## 4 Discussion

### 4.1 Phosphorus fractions, P uptake, and phosphatase enzyme affected by biochar and fishpond amendments

FPS and BR application can alter the P distribution and its availability by changing the soil properties (physiochemical) (Sui et al., 2021; Yang et al., 2021).

In terms of the labile P fractions present in the soil, the NaHCO_3_-Pi fraction denotes adsorbed inorganic P forms that are relatively less labile, while the NaHCO3-Po fraction can be readily mineralized in the soil solution (Bai et al., 2020; Liu et al., 2022). These two fractions are considered the most accessible P pools for supporting plant growth (Gächter and Meyer, 1993; Ahmed et al., 2020). The significant increase in NaHCO_3_-Pi and NaHCO_3_-Po fractions in higher application of simple biochar, modified biochar along with fishpond treatment [(Soil+FPS (35%) + BR (1%, T6) and (soil + FPS (35%) + MBR (1%, T7)], respectively. The results from **Figures 3**, **4** demonstrate an increase in soil P availability, primarily attributed to the direct input of high-available P content from rich nutrient sources such as FPS BR, thus indicating the influence of P legacy (Dietrich et al., 2017; Melia et al., 2019). Another possible reason of higher labile P fraction in T6 is that the FPS amendments are also particularly rich in P due to the large amounts of fish feed, feces, and excretion products that accumulate in the ponds over time (Mehmood et al., 2022). As a result, FPS are often characterized by high levels of total P content (Tammeorg et al., 2022). Furthermore, the increased application of BR in soil has been shown to enhance soil pH, which can contribute to improved P status and availability by reducing the likelihood of P sorption in acidic soil (Ahmed et al., 2021a). Additionally, BR has the potential to enhance the soil microbial environment, leading to enhanced P-related enzyme activities such as phosphatase, which plays a crucial role in the transformation of organic P into inorganic P in soils (Foster et al., 2018; Cao et al., 2023).

The intermediate labile P (NaOH-P_i_ and NaOH-P_o_) fractions are absorbed on the surface of Fe/Al oxides and humic substances (Mehmood et al., 2018). The observed increase in NaOH-Po due to the treatment involving FPS and MBR (T7) can be attributed to the mineralization process, wherein it transforms from a moderately labile form into the more labile NaHCO3-Pi, as indicated by **Figure 3** and supported by the findings of Mnthambala et al. (2022).

HCl_D_-Pi fraction (Apatite P), which is considered as the source of P availability, was higher in higher fishpond (T5 and T7) treatment (**Figure 4**). This could indicate that the fishponds were contributing to higher levels of available P in the soil. Fishponds can be a potential source of P for crop production as fish excreta contains high levels of P. Several studies have reported that fishponds incorporation can increase the availability of P in soils. For example, a study conducted by Potužák et al. (2016); Saviolo Osti et al. (2020) found that fishpond effluent application increased soil apatite P availability, resulting in increased crop yields utilizing the legacy. Similarly, a study by Hepher (1958) reported that the application of fishpond effluent to soils improved soil fertility and increased crop yields. In summary, the statement suggests that the HCl_D_-P_i_ fraction, an indicator of plant-available P in soils, was higher in treatments T5 and T7, which were associated with higher fishponds. This could indicate that fishponds are a potential source of P for crop production and be used as fertilizer.

Furthermore, the formation of phosphate complexes after modified biochar interaction contributed to the increase of HClc-Pi and HClc-Po. According to Rafique et al. (2020), the increase in both residual and labile P pools can be attributed to the precipitation or sorption of P onto recalcitrant organic composites.

Residual P and Total–P represent relatively different trends among the study treatments (**Figure 2**). Residual P is considered as non-labile P and readily available for plant uptake (Sulemana et al., 2021). Higher concentrations for residual P in the higher fishpond, simple and MBR amended treatments might be attributed to the exogenous introduction of BR-carried non-available P (Winkler and Zotz, 2009; Figueiredo et al., 2020). Moreover, FPS contain sources of feed applied, which can be the reason for P accumulation and increases residual P (Mehmood et al., 2022). Some evidence supports the suggestion that simple and MBR can increase soil P levels, including residual P. For example, a study by Ahmed et al. (2021a) found that BR application increased total P and residual P levels in soil, compared with untreated control soils by increasing soil pH and Ca ion concentrations. Similarly, a study by Lyu et al. (2021) reported that BR application increased residual P levels in soil, but the effect depended on the type of biochar used.

The total P concentrations in the higher sediment with MBR incorporation (T7) were higher (**Figure 2**), showing a high level of total P content. Similar results were reported in highly eutrophic sediments incorporated in soils in China (Li and Huang, 2013). Likewise, the highest P concentration in soils is incorporated with the sediments of Dianchi Lake sediment (Li et al., 2007; Zheng et al., 2019). The P in soil and sediment resulted mostly from anthropogenic inputs from fertilizer applications in agriculture soils and later their follow towards lakes and ponds, sewage discharges, and industrial inputs (Ringeval et al., 2014; Meng et al., 2018; Demay et al., 2023). The fact that total P was higher in those treatments with higher FPS amendments may suggest that P resulted from heavy sediments source and MBR with its capability in increasing the pH of the soil and enhancing total P concentrations. It might be also due to the higher Ca-associated P (Apatite) in the soil which comes as alluvial source (Mehmood et al., 2018). The data suggested that total P probably migrated from parent material soil, resulting in higher total P accumulation in response to sediments incorporation (Lu et al., 2020; Mahmood et al., 2022).

The phosphatase enzyme is considered as the indicator of P availability and plays a crucial role in catalyzing the hydrolysis of organic phosphates into inorganic phosphates, which can be taken up by plants (Ahmed et al., 2020).

Our study found that low FPS application and higher MBR application treatment significantly reduced phosphatase enzyme activity and P uptake compared with other treatments while higher FPS lower simple and MBR increased the P uptake and phosphatase activity (**Figure 1**). Our study is in line with the study of Huang et al. (2017), who investigated the effect of BR and sediment amendments on phosphatase enzyme activity and P uptake in the soil and found a decrease in them with higher BR applications. This may also be due to the potential for BR to adsorb and immobilize P, thereby reducing its availability for hydrolysis by phosphatase enzymes and P uptake.

In contrast, the study found that higher FPS and simple and MBR treatments had higher phosphatase enzyme activity, although the difference was not statistically significant (**Figure 1**). This may be due to the fact that FPS are a rich source of organic matter and nutrients, which can stimulate microbial activity and phosphatase enzyme production (Mehmood et al., 2023). These results are consistent with the fact that FPS and BR amendments can improve soil fertility and nutrient availability, thereby enhancing plant growth and P uptake.

Overall, these results suggest that the application of BR and FPS amendments can have a significant impact on phosphatase enzyme activity and P availability in soil and likewise plant uptake, which in turn can affect the availability of P for plant uptake.

### 4.2 Relations between P fractions, P uptake, phosphatase, and metals contents (Ca, Cd, Cr, Zn, and Mg)

Metal contents significantly influence P distribution in soils by binding (Xia et al., 2020). Our results suggest a significant positive relationship between the increase in Zn concentration, organic and inorganic P fractions (NaOH-P_i_, NaOH-P_o_, HCl_C_-P_i_, HClc-P_o_, and residual P), while there was a significant negative relationship between the increase in Zn concentration and available P fractions (NaHCO_3_-P_i_ and NaHCO_3_-P_o_) (**Figure 5**). However, moderate labile fractions (HCl_D_-Pi and residual P) were positively correlated with Zn concentration, indicating that the alkaline environment can promote the removal of Zn reduce the P availability (Ahmad et al., 2018). These findings are in line with the previous research of Nkrumah et al. (2021) that has shown that NaOH-P_o_ fraction can be accumulated and less labile for the plant uptake can effectively decreased by the heavy metal contamination in soils. The reduction in NaHCO_3_-P_o_ with increased concentration of Zn might also be the higher accumulation of moderate labile P fractions (NaOH-Po and HCl_C_-Po) (Golestanifard et al., 2021).

Similarly, Ca concentration did significantly decrease the distribution of labile inorganic and organic P (NaHCO3-Pi and NaHCO_3_-Po), increasing the moderate and recalcitrant or nonlabile organic P fractions. This indicates that Ca may not have a significant effect on the removal of P from soil to make it labile; however, it promotes the fixation (Mahmood et al., 2022). Pearson correlation analysis (**Figure 7**) gives positive relationship between moderate labile P fractions (HCl_D_-P_i_ and HClc-P_i_) and confirms soils a large amount of Ca-associated P (Apatite) (organic and inorganic) that is presented, which is not readily available for the plant uptake (Ahmed et al., 2020). Likewise, cadmium (Cd) and P are shown to have a complex and negative relation (**Figure 7**). On the one hand, Cd in higher amount can have negative impacts on availability of P and its cycling in soil system, while on the other hand, Cd can affect P uptake and toxicity in plants and other organisms (Kovačević et al., 2021; Maqbool et al., 2022). For example, a study by Cullen and Sherrell (2005) found that Cd contamination decreased soil P availability and inhibited the activities of enzymes involved in P cycling. Similarly, a study by Yang et al. (2015) found that Cd exposure reduced the abundance of microbial groups involved in organic P mineralization and decreased soil P availability.

Overall, these findings suggest that Cd and P have complex interactions in the environment and that the effects of Cd on P availability and cycling, as well as the effects of P uptake, should be taken into account in environmental management and risk assessment.

Cr and Mg concentration had a significant effect on the distribution of organic P fractions. Labile P fractions (NaHCO_3_-Pi, NaHCO_3_-Po, and NaOH-Po) showed a significant decrease with an increase in Cr concentration, while NaOH-Po showed a significant increase (**Figure 6**). However, previous research has suggested that Mg may enhance the formation of metal hydroxides, which can adsorb and remove P from soil but stimulating its pH and environmental conditions on the binding sites (Ahmed et al., 2020). In contrast, Cr may interact with the surface charges of metal hydroxides and affect their adsorption capacity for P (Shaheen and Iqbal, 2018).

Overall, the results suggest that balance application of BR and FPS is necessary to maintain initial concentrations of Cr and Mg, so that P availability can be maximized for plant uptake.

The study found that higher rates of applied BR and FPS resulted in significant differences in P uptake and phosphatase enzyme activity compared with lower rates (**Figure 1**). There was a positive correlation (**Figure 7**) between phosphatase enzyme activity and P uptake, suggesting that higher BR levels increased P availability. FPS were also found to be a rich source of nutrients that enhance P availability and uptake (Mehmood et al., 2021).

Past studies have also shown that the application of BR and sediments increases total P and leads to more P fixation (Tesfaye et al., 2021). The study found that the higher BR and sediment application levels resulted in higher levels of total recalcitrant or non-labile P compared with lower levels and showed a positive correlation with (Ca) calcium-associated fractions with P uptake and Olsen P (**Figure 7**). The study also found BR and sediments application increased total and accessible P contents compared with other lower applied rates, indicating that BR and sediments application at different levels caused interactive effects on soil available P concentration. This may be due to increased microbial activities that increased the amount of accessible Pi and reduced the number of distinct P forms fixed in soils (Ahmed et al., 2021b; Mahmood et al., 2022).

## 5 Conclusion

This study aimed to assess the impact of BR and/or FPS on soil P fractions and availability, their consequences for P uptake by maize plants. The results indicate that the addition of BR and FPS significantly increased P availability. Both solo and combined applications of BR and FPS significantly improved soil P fractions and had a profound influence on plant P uptake. Specifically, the application of 1% BR in combination with 35% FPS showed notable effects on the moderate labile P fraction and phosphatase enzyme activity. Soil available P content was consistently lower under higher BR treatments combined with FPS, highlighting the importance of applying optimal rates of BR and FPS to enhance P availability while minimizing P fixation and residual P loss, which ultimately maintains plant P uptake. Our study provides valuable insights into the possibility of applying BR along with FPS as an effective strategy to improve P use efficiency, which ultimately would maintain sustainable food production.

## Data availability statement

The original contributions presented in the study are included in the article/supplementary material. Further inquiries can be directed to the corresponding authors.

## Author contributions

Conceptualization: MM. Methodology: WL and XZ. Validation: MM and WL. Formal analysis: MM. Investigation: YW. Data curation: AE. Writing—original draft preparation: MM, WA, SM, and AA. All authors have read and agreed to the published version of the manuscript.
